# Valorization of Agricultural By-Products (*Fragaria vesca*) through the Production of Value-Added Micro/Nanostructures Using Electrohydrodynamic Techniques

**DOI:** 10.3390/foods13081162

**Published:** 2024-04-11

**Authors:** Ana Francisca Couto, Berta N. Estevinho

**Affiliations:** 1 LEPABE—Laboratory for Process Engineering, Environment, Biotechnology and Energy, Department of Chemical Engineering, Faculty of Engineering, University of Porto, Rua Dr. Roberto Frias, 4200-465 Porto, Portugal; 2 ALiCE—Associate Laboratory in Chemical Engineering, Faculty of Engineering, University of Porto, Rua Dr. Roberto Frias, 4200-465 Porto, Portugal

**Keywords:** circular economy, electrohydrodynamic, *Fragaria vesca*, microencapsulation, quercetin, zein

## Abstract

An innovative approach for the production of bio-micro/nanostructures with high-value compounds from agricultural by-products was studied. This research aimed to valorize bioactive compounds existing in the by-products of the plants of *Fragaria vesca* (wild strawberry). The particle characteristics, morphology, size, release properties, and antioxidant activity of micro/nanostructures containing the extract of by-products of the plants of *Fragaria vesca* or quercetin (one of the main polyphenols in the plant) were analyzed. The electrohydrodynamic (EHD) technique was utilized for encapsulation. The results showed that the morphology and size of the structures were influenced by the concentration of zein, with 10% *w/v* zein concentration leading to irregular and non-uniform nanostructures, while 20% *w/v* zein concentration resulted in a mixture of microparticles and thin fibers with an irregular surface. The type and concentration of the core material did not significantly affect the morphology of the micro/nanostructures. *In vitro* release studies demonstrated the controlled release of the core materials from the zein micro/nanostructures. The release profiles were analyzed using the Korsmeyer–Peppas and Weibull models, which provided insights into the release mechanisms and kinetics. The most relevant release mechanism is associated with “Fickian Diffusion”. The antioxidant activity of the structures was evaluated using an ABTS radical-scavenging assay, indicating their potential as antioxidants. In conclusion, the EHD technique enabled the successful encapsulation of *Fragaria vesca* by-product extract and quercetin with zein, resulting in micro/nanostructures with different morphologies.

## 1. Introduction

The valorization of by-products and the maximum utilization of raw material constitute highly relevant topics in the EU and worldwide, as the reduction in food waste production is one of the goals of the United Nations for 2030, to achieve a more sustainable world [1].

Agricultural and food wastes (agri-food waste) should be thought of as sources of plentiful value-added products, and they should be targeted to achieve a zero-waste economy. Around 89 million tons of food is wasted annually in the European Union [2]. Therefore, agro-industrial activities are characterized by high rates of generation of organic waste, for example, from fruits such as peels, trimmings, stems, seeds, fruit malformations, etc., which can sometimes represent more than 50% of a fresh fruit [3,4]. The agri-food wastes are high in volume and with low-value materials [2]. Therefore, the circular economy that allows the closing of cycles in the waste value chain and the generation of value from these by-products is important [5]. These kinds of by-products still have the potential to be used in other economical and industrial sectors, due their physical, chemical, and microbiological characteristics [6,7,8].

The circular economy applied to the agro-industrial activities promote the generation of strategies that mitigate environmental problems and, at the same time, contribute to obtain value from residues [9,10]. Therefore, in this study, the by-products from wild strawberry were used to prepare innovative add-value formulations, to be applied in different fields such as food and nutraceuticals, considering their health benefits [11].

Wild strawberry, scientifically known as *Fragaria vesca*, is a member of the Rosacea family and is usually found in Europe, Asia West of the Urals, and North America [12,13,14]. This plant species has a rich history in traditional medicine, where it has been widely recognized for its anti-inflammatory properties and its potential in the treatment of gastrointestinal disorders [4,11,12,15]. *F. vesca* leaf extract emerges as an important ingredient, being the source of polyphenols [15,16,17]. The main phenolic compounds present in this plant are flavonoids, proanthocyanidins, ellagitannins, phenolic acids, volatile oils, catechins, methyl salicylate, ellagic acid, borneol, and also trace amounts of alkaloids [13,17,18]. Also, the protective effect on the skin provided by *F. vesca* leaves is attributed to the abundance of diverse polyphenolic compounds and their inherent antioxidant properties [4,18,19].

In this context, the *Fragaria vesca* by-product extract can present several health benefits if correctly incorporated into food or nutraceuticals [13]. Microencapsulation is a technique used to create a physical barrier between a sensitive compound, like the phenolic compounds in the extract, and the external factors [20,21]. This approach has the potential to enhance the efficiency of various components, enabling the modification of numerous food products. It leads to the enhancement and introduction of superior and novel qualities, which have beneficial effects on the human body [20,21].

Electrohydrodynamic techniques (EDHs) were selected as the encapsulation method for the present study due to their inherent advantages like no need for high temperatures and extreme pH conditions, unlike other traditional encapsulation methods [22,23]. EHD processes utilize electrostatic fields of high voltage to electrically charge the surface of a polymer solution jet [22,23]. The electrohydrodynamic techniques (EDHs) are based on the ability of an electric field to deform the interface of the droplet and get droplets in the range of a micrometer or nanometer, depending on the parameters that need to be controlled. This principle is based on the theory of charged droplets which establishes that if an electric field is applied to a droplet, the electric charge generates an electrostatic force in the droplet, known as the Coulomb force, which competes with the force of cohesion in the droplet. Thus, when a critical voltage is applied, the Coulombic repulsion of the charges overcomes the solution droplets’ surface tension, and then, the jet is ejected. A limit, named the Rayleigh limit (LR), exists in the determination of the rupture of the droplet. This limit is when the surface tension of the drop is overcome by the electrostatic force [22,23]. Two similar methods can be employed in the EDH processes: electrospinning, which results in the production of electrospun fibers, and electrospraying, which similarly creates micro/nanoparticles [21,23]. The key difference between these two approaches lies in the polymer concentration, with electrospraying occurring when the concentration is low enough to destabilize the jet and form fine-charged droplets [24]. However, due to the complex nature of these processes, there are several conditions and parameters that can be adjusted to obtain microstructures with different characteristics.

The decision to use zein as the encapsulating agent in the present study took into account the previous scientific studies and an assessment of its advantages and disadvantages as a biopolymer [20,21]. Zein is a prolamin protein derived from the endosperm of corn, constituting a significant portion of the total protein content in corn. Its hydrophobic properties, low moisture absorption, high thermal resistance, and oxygen barrier capabilities make it a highly desirable material for processing and encapsulating various compounds [25]. Zein is a biodegradable and biocompatible biopolymer, and it is soluble in ethanolic solutions, namely, 70% ethanol. Zein has been extensively employed in diverse industries, including pharmaceuticals and food, as it has received approval from the Food and Drug Administration (FDA) as a generally recognized as safe (GRAS) product.

This study is innovative and relevant because it allows the utilization of agro-industrial by-product from *Fragaria vesca* as a food additive, and it also allows the promotion of the circular economy. To the best of our knowledge, no studies on microencapsulation have been performed with the extracts of the *Fragaria vesca* by-products and by electrohydrodynamic methods. Thus, the primary aim of this research was to assess the particle characteristics produced using the natural *Fragaria vesca* by-product extract and quercetin (one of the main polyphenols in the plant) and to compare the morphology, size, and release properties of the microstructures obtained with different zein and core concentrations. Additionally, the antioxidant activity of the micro/nanoparticles was evaluated.

## 2. Materials and Methods

### 2.1. Reagents and Solutions

*Fragaria vesca* leaves were produced in greenhouses in the north of Portugal, close to Vila do Conde. For the polyphenol’s extraction, 99% ethanol was used (Cat. No. 71023001.00591) and was provided by Valente e Ribeiro LDA. Quercetin (Cat. No. Q4951-100G, Lot: SLCK5305, CAS: 117-39-5) was used as a standard/polyphenol model and was obtained from Sigma-Aldrich (St. Louis, MO, USA).

For the encapsulation process, zein (grade Z3625) was used as an encapsulating agent, and its powder was purchased from Sigma-Aldrich (St. Louis, MO, USA) and used as received, without further purification. The 70% aqueous ethanol solution was purchased from VWR BDH-chemicals and used as a solvent. The selection of the encapsulating agent type and concentration was based on a previous study [21].

For the release assays, deionized water, i.e., Milli Q water, with the resistivity of 18.2 MΩ/cm at room temperature (~23 °C), was used for the *in vitro* release assays and the construction of calibration curves.

For the antioxidant activity experiment, 2.2′-Azino-bis(3-ethylbenzothiazoline-6-sulfonic acid) diammonium salt (ABTS) (MW: 548.68 g/mol), (±)-6-Hydroxy-2,5,7,8-tetramethylchromane-2-carboxylic acid (Trolox) (MW: 250.29 g/mol), and potassium persulfate were used and were purchased from Sigma-Aldrich (St. Louis, MO, USA).

### 2.2. Fragaria vesca Leaf Extract Production

According to the extraction method used, a complex mixture of polyphenols is derived from the *Fragaria vesca* leaves. The most appropriate procedure for isolating polar chemicals, such as flavonoids, is solvent extraction, supported by the use of ultrasound, a technology that has small effects on the end product [26,27]. Thus, in the current study, the leaves were subjected to an alcoholic extraction in an ultrasonic bath [27].

Firstly, 4.0 g of *Fragaria vesca* leaves were crushed using a mortar and a pestle and mixed with 80 mL of 99% ethanol and 20 mL of deionized water. The resultant mixture was stirred at room temperature for one hour at 560 rpm. After, the solution was subjected to two 30 min intervals in an ultrasonic bath (Ultrasound–Elma S30H, Elma-sonic, Singen, Germany), with a total treatment of one hour. The solution was centrifuged (Centromix S—549) for 20 min, at 4000 rpm. Lastly, the supernatant was collected and stored in the freezer.

### 2.3. Micro/Nanostructure Production

**Figure 1:**
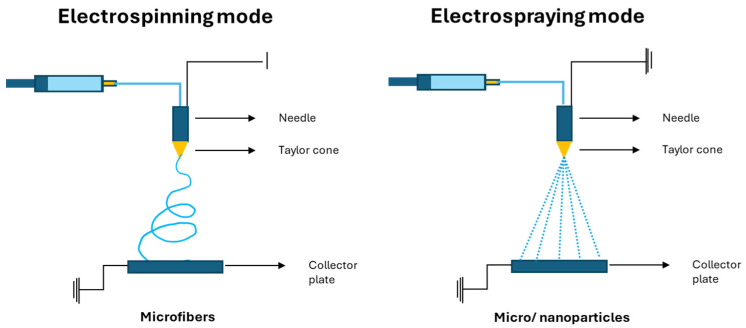
Schematic illustration of electrohydrodynamic technologies: electrospinning and electrospraying modes.

The EHD was performed for different formulations prepared with different solutions (Figure 1). Three different types of solutions were prepared, i.e., the zein solutions, the zein + quercetin solutions, and the zein + extract solutions. All solutions were prepared under similar conditions. Zein was dissolved in aqueous ethanol and stirred at room temperature until completely dissolved. Solutions with different concentrations were prepared, i.e., 10% and 20% *w/v* (weight of zein/volume of solvent). Studies with 30% *w/v* zein were also performed at a preliminary stage, but electrospun fibers were produced, and thus, these formulations were excluded from the following stages, considering our objective which was to produce particles to simplify its manipulation and incorporation into food products. The chosen zein concentrations are in accordance with the preliminary optimization of the method reported in previous studies [21,28]. Two different core materials were used, i.e., the extract and quercetin (one of the main polyphenols in the plant). Both were prepared in aqueous ethanol and added to the zein solution to achieve 1% and 5% total solid content (weight quercetin/(weight quercetin + weight of zein)). The concentrations and conditions are listed in Table 1.

Different microstructures were generated using an electrospinning/electrospraying experimental setup provided by Spraybase^®^ (Dublin, Ireland) that was equipped with a variable high-voltage (0–20 kV) power supply. An electric pump and a stainless-steel needle (22 G) positioned 5 cm distant from a metal collecting plate were used to pump the solutions at a steady-state flow rate (Figure 1). During the procedure, a voltage of 20 kV was applied. The flow rate used for each sample is shown in Table 1. The optimal conditions were considered, and the process occurred at room temperature (approximately 22 °C). The resulting micro/nanostructures were scraped from the collector and placed in plastic containers.

### 2.4. Conductivity of the Zein Solutions

The zein polymer solutions (10%, 20%, and 30% *w*/*v*) were characterized in terms of electrical conductivity. Two solutions, containing quercetin and the *Fragaria vesca* leaf extract, were also evaluated. The conductivity was evaluated with a conductivity meter (MultiLab 540, WTW, Straubing, Germany). The conductivity analysis was performed at room temperature (~22 °C) using a 20 mL sample for each analysis.

### 2.5. Microstructure Morphology

Scanning electron microscopy, SEM, Fei Quanta 400 FEG ESEM/EDAX Pegasus X4M (Eindhoven, The Netherlands), was used to analyze the morphology and size of the microstructures in different parts of the samples at Centro de Materiais da Universidade do Porto (CEMUP), Porto, Portugal. The magnifications used in each sample were 100×, 1000×, 10,000×, 30,000×, and 50,000×; the beam intensity (HV) was 15.00 kV. Different areas of the samples were observed in order to guarantee their homogeneity.

The mean particles sizes were measured using the IMAGEJ software (v1.54d). More than 200 particles were analyzed for each sample.

### 2.6. In Vitro Release Studies

The *Fragaria vesca* extract (evaluated in terms of quercetin) and quercetin release profiles of zein microstructures were determined in deionized water, at room temperature, to simulate commonly used industrial formulations. The profiles were obtained using NanoDrop One-C spectrophotometer (Thermo Fisher Scientific, Norristown, PA, USA) by a continuous absorbance measurement (376 nm) until the value stabilized.

Due to its representativeness in the extract composition, quercetin was employed as the standard compound. To validate the method, a calibration curve was obtained, using 10 standard solutions, with quercetin concentrations ranging from 0.0001 to 0.001 mg/mL. The absorbance was measured in triplicate, and its average was represented as a function of the standard concentrations. The coefficient of variation, the limit of detection (LOD), and the limit of quantification (LOQ) were determined and are presented in Table 2.

The release assays were performed by placing an amount of sample in deionized water. Water was used as a solvent to simulate commercial aqueous formulations, which is a standard medium used for food products. The amount of powder used in the release tests was estimated by a mass balance, considering the quantification limits of the calibration curve. All the absorbance measurements were evaluated with agitation, at room temperature, and in triplicate.

In order to evaluate the kinetic behavior of the core release, two different models were adjusted to the release profiles, as described in Estevinho and Rocha, 2017 [29]. The Korsmeyer–Peppas, Equation (1), and Weibull, Equation (2), models are popular for characterizing active ingredient release and optimizing process parameters to produce structures with specified features [29].

(1) $$\frac{Q_{t}}{Q_{\infty }}=K_{k}t^{n}$$

(2) $$\frac{Q_{t}}{Q_{\infty }}=1-e^{{-\left(\frac{t-t_{0}}{\tau_{d}}\right)}^{\beta }}$$

where *Qt/Q∞* is the fraction of the active compound released until time *t*, *K_K_* is the Korsmeyer constant, *n* is the release exponent (a parameter that defines the release mechanism), and β is the shape parameter of the curve [29].

### 2.7. In Vitro Antioxidant Activity Experiment

Several methods and adaptations can be found in the literature; however, the ABTS and DPPH methods are the most popular assays of antioxidant activity determination [30,31]. The authors considered the ABTS method for this study, considering preliminary results. The antioxidant activity of the produced microstructures was evaluated using an ABST* radical-scavenging assay [28]. The procedure was based on the method described by Re et al., 1999 [32]. Briefly, 7.4 mM ABTS aqueous solution was prepared and mixed with 2.6 mM potassium persulfate aqueous solution, and it was incubated for 16 h in the dark. For the preparation of the ABTS* solution, 60 mL of pure ethanol was added to 1 mL of the solution incubated overnight. The samples (150 µL) were mixed with the ABTS* solution (2850 µL) and allowed to react for 2 h, in dark conditions. The analysis was made at 734 nm using the NanoDrop One-C spectrophotometer (Thermo Fisher Scientific, USA).

### 2.8. Statistical Analysis

All the analytical determinations were performed in triplicate, and the results were expressed with standard deviations associated with the measures. The results of statistical significance were analyzed (at a level of significance of *p* ≤ 0.05) by single-factor analysis of variance (ANOVA) and Tukey’s test.

## 3. Results and Discussion

In the present study, the electrospinning technique was used to produce micro/nanostructures with zein as an encapsulating agent and two different core materials (*Fragaria vesca* leaf extract and quercetin) for food applications. Additionally, empty microstructures were also produced. The morphology, antioxidant capacity, and release profiles of the prepared microstructures were evaluated and are discussed in the next subsection.

### 3.1. Characterization of the Microstructures

**Figure 2:**
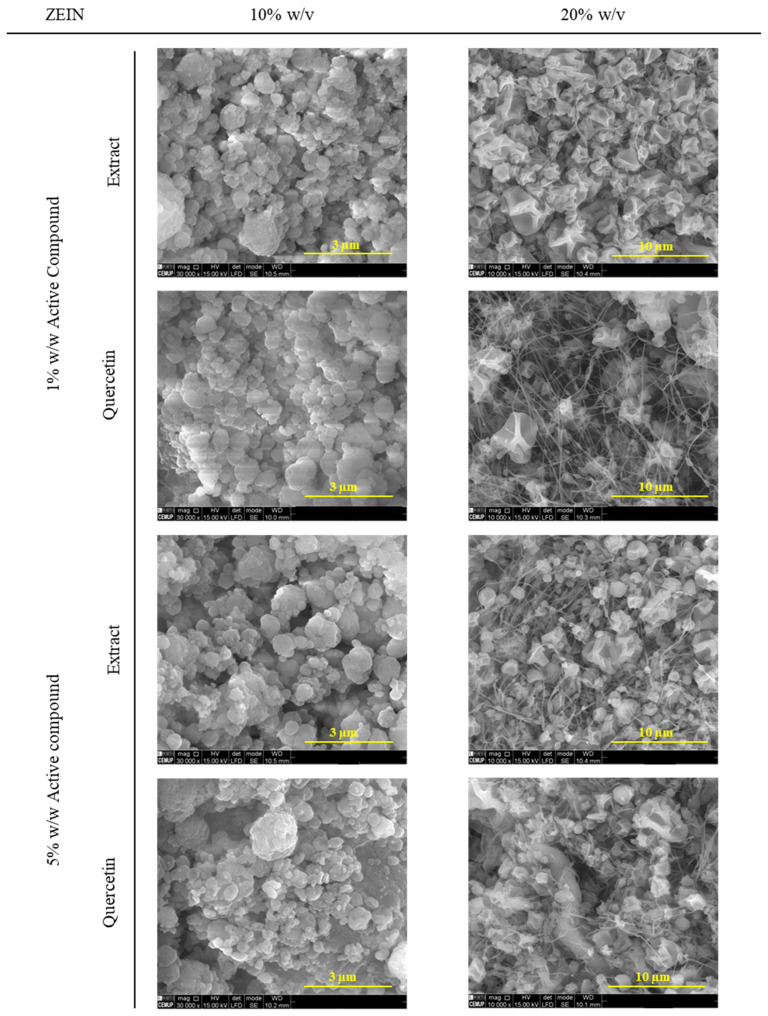
SEM images of microstructures containing the different concentrations of zein, extract, and quercetin. Magnification of 30,000× for samples with 10% *w/v* zein and 10,000× for samples with 20% *w/v* zein, a beam intensity (HV) of 15.00 kV, and a distance between the sample and the lens (WD) of less than 10.5 mm.

Microstructures containing *Fragaria vesca* extract and quercetin were evaluated using scanning electron microscopy (SEM), refer to Figure 2. Two different zein concentrations (10 and 20% *w*/*v*) were selected in order to obtain micro/nanoparticles, considering the stability and the applicability in the food industry.

In previous studies, it was concluded that lower concentrations of zein make the process very slow and that higher concentrations of zein provoked the formation of fibers [20,21,33].

In an initial approach, a notable difference in morphology is evident between the lowest zein concentration studied, 10% *w*/*v*, and the highest concentration studied, 20% *w*/*v*. At 10% zein concentration, the formed capsules exhibit a wrinkled appearance and display some heterogeneity in size. This observation suggests that the lower concentration may result in an electrospraying process. On the other hand, for the concentration of 20% *w/v,* the formation of fibers begins and is associated with the electrospinning process. Therefore, the concentration of zein of 20% *w/v* must be associated with the transition stage between electrospraying and electrospinning.

**Figure 3:**
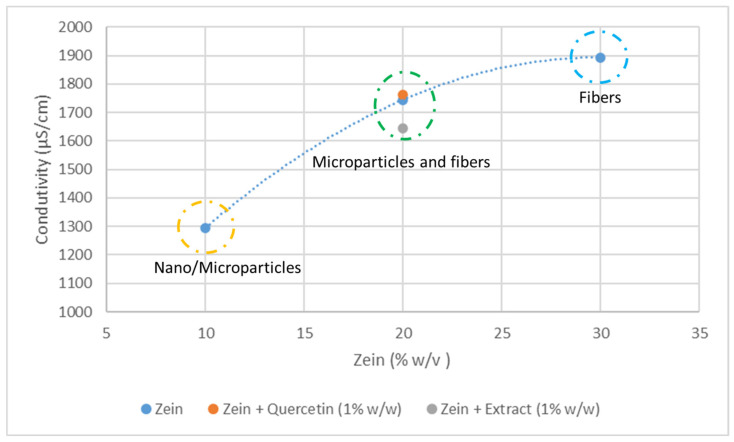
Conductivity of solutions containing the different concentrations of zein, extract, and quercetin.

Therefore, at the higher zein concentration of 20% *w*/*v*, a distinct pattern emerges. The microstructures formed consist of a mixture of microparticles and thin fibers. These microparticles, although less wrinkled compared to the capsules formed at 10% *w*/*v* zein concentration, still exhibit heterogeneity in size. Curiously, the microparticles demonstrate an irregular surface, similar to possessing an outer layer that has undergone a collapsing phenomenon during the solvent evaporation process [34]. This unique characteristic suggests a potential structural transformation occurring during the fabrication process, possibly resulting from the interplay between zein concentration, solvent evaporation, and polymer self-assembly. The observed mixture of fibers and microparticles in the higher concentration samples is likely attributed to the increased resistance of these solutions to be drawn towards the collector during the electrohydrodynamic process or may be influenced by the electrical conductivity of the solution [35]. The electrical conductivity of the zein solution was evaluated and compared with the solutions containing quercetin and extract (Figure 3).

The conductivity reveals the charge density on a jet and, consequently, its elongation [36]. The electrical conductivity increases with the zein concentration, which means that there is an increase in ionizable groups present in the solution [37]. The fibers appear associated with higher concentrations of zein and, consequently, higher values of conductivity. This result is in concordance with those of other authors, namely, Coelho et al. (2022) [33].

On the other hand, the addition of quercetin to the zein solution does not affect the electrical conductivity significantly. In the case of the addition of the *Fragaria vesca* leaf extract to the zein solution, the conductivity decreases slightly. The decrease in the conductivity is also associated with the decrease in the number of fibers that appear in the SEM images of the sample containing the extract.

Moreover, according to Table 3, the average size of the particles increases for higher concentrations of zein. For a concentration of zein of 10% *w/v*, it is possible to obtain nanoparticles, and for a zein concentration of 20% *w/v*, we obtain microparticles around 1 µm. The type of active compound does not have any influence on the size of the micro/nanoparticles.

It is noteworthy that the morphology of the microstructures remains unaffected by the type or the concentration of the core material, indicating that, in the context of this study, the concentration of the encapsulating agent plays a crucial role in determining the formation of diverse structures through electrospinning/electrospraying.

Another parameter evaluated was the polydispersity index, and it is used to describe the degree of non-uniformity of the size distribution of particles [38,39]. In a preliminary evaluation, the microparticles prepared, in general, can be considered as monodisperse nano/microparticles.

The observations made in our study regarding the morphology and formation of microstructures using electrohydrodynamic techniques for encapsulating *Fragraria vesca* leaf extract and quercetin align with the findings reported by Bruni et al., 2020 [40]. Both studies emphasize the influence of concentration on the formation of distinct structures. Our research demonstrates that varying zein concentrations lead to different microstructure morphologies, while Bruni et al. highlight the successful encapsulation of yerba mate extract within electrospun protein fibers [40].

### 3.2. In Vitro Release Assays

The functionality of active ingredients can be significantly enhanced through microencapsulation, as it enables their safeguarding and targeted release based on desired conditions. This, in turn, creates opportunities for their utilization across a wide range of products [27]. The release mechanisms of encapsulated substances depend on factors such as the type of encapsulating agent, the method of preparation, and the environmental conditions during release. These mechanisms can involve individual processes or their combination, including diffusion, degradation, biodegradation, gel formation, swelling, melting, and osmosis [29].

**Figure 4:**
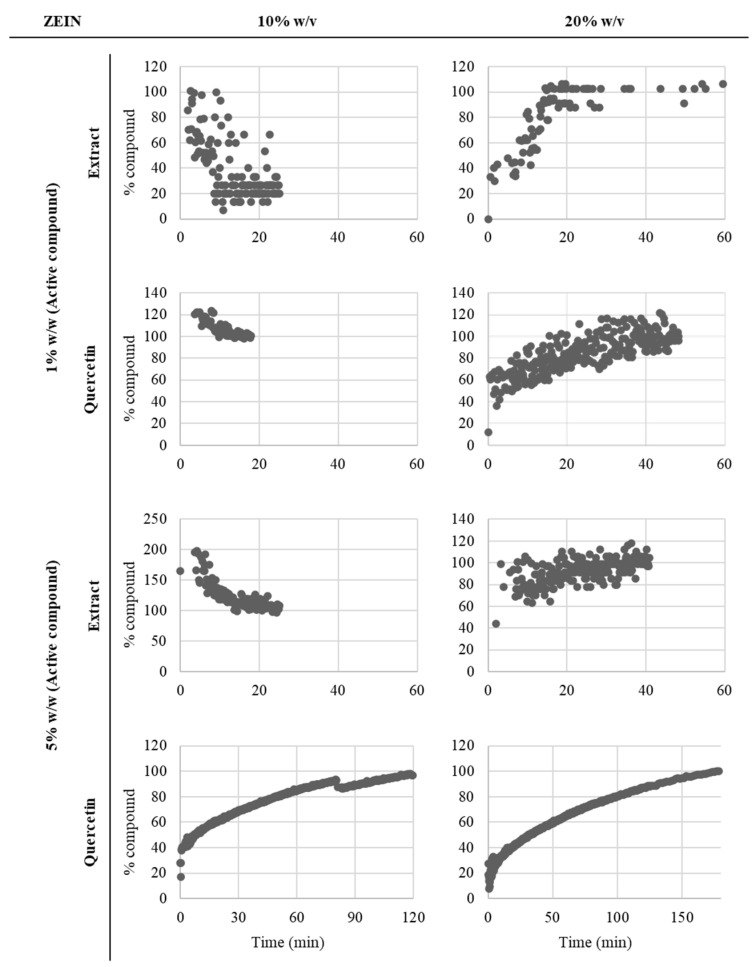
*In vitro* release profiles, % of extract or quercetin normalized by the total amount released, in deionized water of the produced microstructures.

The release profiles associated with the core release from the microstructures are depicted in Figure 4.

The release profiles of the encapsulated samples exhibit distinct behaviors, highlighting the influence of different formulations on the release kinetics. In general, the samples containing extract have more unstable and complex release profiles, but this can be justified because the extract is a mixture of different active compounds that also make the analysis of the release profile (based on the polyphenol quercetin) difficult.

Immediate release is observed in the samples prepared with 10% *w/v* of zein (10Z1Q, 10Z1EX, and 10Z5EX) except for the sample prepared with 5% *w*/*w* of quercetin. The immediate release is also associated with the morphology of particles and have already been described by the authors of studies involving other release systems prepared with zein [20,21,28]. In contrast, the samples containing 20% *w/v* zein exhibit better controlled release profiles, suggesting better encapsulation capacity and control. The structures prepared with 20% *w*/*w* of zein have several fibers in their constitution, which influence their release behavior, provoking release over time. On the other hand, the samples containing quercetin have higher conductivity than the ones prepared with the extract and have more fibers in their composition, which can provoke the delay of the release. For this reason, the samples prepared with quercetin have slower releases compared with the ones prepared with the extract.

The sample with 20% *w/v* zein and 1% *w*/*w* extract initially displays a burst release, followed by stabilization within approximately 20 min. Similarly, the sample with 20% *w/v* zein and 1% *w*/*w* quercetin demonstrates a controlled release pattern without an initial burst, reaching stabilization after around 40 min. Notably, the sample with 20% *w/v* zein and 5% *w*/*w* extract exhibits a rapid release, reaching 100% release within 30 min. Furthermore, the release profiles of the samples 10Z5Q and 20Z5Q exhibit relatively slower release kinetics, requiring approximately 120 and 160 min, respectively, to reach the complete release of the core material. These extended-release times suggest that the higher concentration of quercetin also influences the encapsulation process, leading to a better controlled and sustained release over a longer duration. However, the samples prepared with 10% *w/v* of zein and with 5% *w*/*w* of quercetin do not have fibers in their composition and have some characteristics that allow a better controlled release profile instead of an immediate release.

In conclusion, the release profiles of the encapsulated samples indicate that the zein concentration plays a critical role in determining the encapsulation efficiency (close to 100% for all the samples) and release kinetics. Higher zein concentrations, such as 20% *w*/*v*, result in better controlled release patterns, while lower concentrations exhibit immediate release and reduced encapsulation efficiency. Additionally, the type and concentration of the core material, such as quercetin and extract, also impact the release behavior, with different formulations exhibiting variations in release kinetics. These findings contribute to our understanding of the factors influencing encapsulation and release, offering valuable insights for the design and optimization of microstructures in electrohydrodynamic techniques for controlled release applications.

Chaumun et al., 2020, also studied the evaluation of release profiles of encapsulated extracts. *Laurus nobilis* L. extract was encapsulated in different agents by spray-drying. Similar to the present study, higher concentrations of an encapsulating agent resulted in slower and better controlled release kinetics when compared to lower concentrations [27].

To analyze the release kinetics, two kinetic models, Weibull and Korsmeyer–Peppas models, were adjusted to the data. The parameters obtained by the adjustment are presented in Table 4.

However, it was observed that the models could not be fitted to some samples due to their fast or immediate release behavior. The correlation coefficients obtained from the model fittings ranged from 0.283 to 0.985. It was observed that the samples with slower and better controlled release exhibited higher correlation coefficients, suggesting a better fit of the models to these release profiles, namely, for the two samples containing quercetin 5% *w*/*w* (10Z5Q and 20Z5Q). However, it is important to note that the overall correlation coefficients obtained in this study were relatively low compared to other similar studies. This may indicate that the release behavior of the encapsulated microstructures is complex and not fully captured by the selected models, suggesting the need for the further investigation and refinement of the kinetic models for a better understanding of the involved release mechanisms.

By applying the Korsmeyer–Peppas model, two parameters, *K_k_* and *n*, were obtained. The parameter *n*, known as the release exponent, provides insights into the mechanism responsible for the release of the core material [29]. Analyzing the obtained *n* values, all the samples present a *n* lower than 0.45. This indicates that, for the samples where it was possible to adjust the models, the release of the core material is associated with “Fickian Diffusion”, which is characterized by a better controlled and diffusion-based release.

Meanwhile, the Weibull model yielded the parameter *β*, which is related to the shape of the release curve. These parameters offer valuable information regarding the release characteristics and can contribute to the understanding of the underlying mechanisms governing the release process [41]. The value of the parameter “β” was less than one for all samples. Thus, the release curves showed an exponential shape but with a steeper increase when compared to the ones with *β* = 1.

### 3.3. Antioxidant Capacity Assessment (ABTS Method)

**Figure 5:**
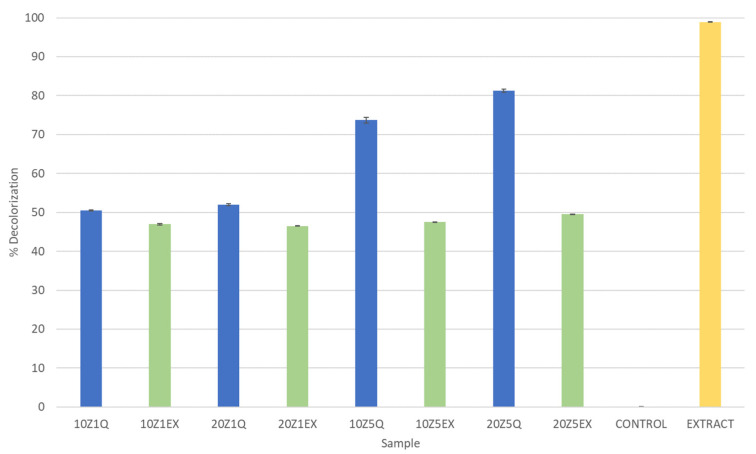
Antioxidant capacity expressed in % of decolorization for all produced microstructures.

The antioxidant capacities of the *F. vesca* leaf extract and quercetin loaded on the zein microstructures were evaluated by the ABTS method. The analysis was performed, and the results are presented in Figure 5. The results are expressed as the percentage of decolorization.

The decolorization percentages observed in the ABTS assay ranged from 46.5% to 81.3%. Analyzing the results, it becomes evident that the samples containing 5% of the active compound and those loaded with quercetin exhibited the highest decolorization percentages. Additionally, a distinct pattern emerges when comparing the samples loaded with the extract to those loaded with quercetin. Regardless of the zein concentration, the former demonstrated lower decolorization percentages, suggesting that the antioxidant activity of the extract, when encapsulated in zein through electrohydrodynamic techniques, is diminished.

Furthermore, when comparing the values obtained for the samples with the original extract, it is important to note that a portion of the antioxidant activity is lost during the encapsulation process or storage of the samples. In some instances, more than half of the antioxidant activity appears to be compromised.

These findings emphasize the impact of different factors, such as the type of compound used (the extract or quercetin) and the encapsulation process, on the observed antioxidant activity in the ABTS assay. Moreover, the results indicate that further investigation is required to understand the factors responsible for the loss of antioxidant activity during encapsulation or storage.

In one study conducted by Faki et al., 2019 [42], the authors investigated the impact of the electrospinning process on the total antioxidant activity of electrospun nanofibers loaded with grape seed extract. Both studies evaluated the antioxidant activity using different encapsulating agents and core materials. In our study, zein was used as the encapsulating agent, while Faki et al. utilized polyvinyl alcohol (PVA) as the polymer matrix for electrospinning. The core materials also differed, but both were extracts. Faki et al. examined the grape seed extract [42]. Regarding the antioxidant activity results, both studies reported a decrease in antioxidant capacity after encapsulation. In our study, the decolorization percentages obtained in the ABTS assay ranged from 47.5% to 81.3%. Similarly, Faki et al. observed a reduction in the total antioxidant activity of the electrospun nanofibers compared to the original grape seed extract. These findings suggest that the encapsulation process, regardless of the encapsulating agent or the core material, may lead to a loss of antioxidant activity.

Thus, micro/nanostructures with interesting properties were produced and can be used in the food industry, for incorporation into food products, or consumed directly as a nutraceutical powder. The produced micro/nanostructures allow one supplementation in terms of polyphenols of 10–50 mg polyphenols/g of produced powder (micro/nanostructure). According to the literature, consuming more than 650 milligrams per day of polyphenols reduces death risk [43,44]. However, there are some concerns regarding polyphenol fortification and supplementation that should be handled with moderation [45,46].

## 4. Conclusions

Bio-micro/nanostructures with high-value compounds from *Fragaria vesca* (wild strawberry) by-products were produced.

The results showed that the morphology and size of the structures were influenced by the concentration of zein, with 10% *w/v* zein concentration leading to irregular and non-uniform nanostructures, while 20% *w/v* zein concentration resulted in a mixture of microparticles and thin fibers with an irregular surface. On the other hand, the type and concentration of the core material did not significantly affect the morphology of the micro/nanostructures.

The samples containing 20% *w/v* zein exhibit better controlled release profiles, suggesting better encapsulation capacity and control. Moreover, the samples prepared with quercetin have a slower release compared to the ones prepared with the extract.

In conclusion, the release profiles of the encapsulated samples indicate that the zein concentration plays a critical role in determining the release kinetics. The release of the core is associated with “Fickian Diffusion” characterized by a better controlled and diffusion-based release. Some antioxidant activity is lost during the encapsulation process or the storage of the samples.

Thus, the produced micro/nanostructures allow one supplementation in terms of polyphenols of 10–50 mg polyphenols/g of produced powder (micro/nanostructure).

These findings contribute to the potential application of these encapsulated micro/nanostructures in various fields, such as food and nutraceuticals, for their health benefits and improved functionality.

## Figures and Tables

**Table 1:** Description of the conditions tested in electrospinning process for the samples containing quercetin or *Fragaria vesca* leaf extract.

Biopolymer	Biopolymer Concentration (% *w*/*v*)	Core Material	Total Solid Content (% *w*/*w*)	Flow Rate (mL/h)	Sample Code
Zein	10	Quercetin	1	1.0	10Z1Q
			5	1.0	10Z5Q
		Extract	1	1.0	10Z1E
			5	1.0	10Z5E
	20	Quercetin	1	2.0	20Z1Q
			5	2.0	20Z5Q
		Extract	1	2.0	20Z1E
			5	2.0	20Z5E

**Table 2:** Parameters of the analytical method.

Compound	Solvent	Equation	R2	LOD (mg/mL)	LOQ (mg/mL)
Quercetin	Deionized Water	y = 59.67x + 0.005	0.998	0.000043	0.000144

**Table 3:** Average size of the micro/nanoparticles prepared with different concentrations of zein, extract, and quercetin.

Active Compound		Zein (% *w*/*v*)	Particle Average Size (µm)
1% *w*/*w*	Extract	10	0.22 ± 0.02
		20	1.87 ± 0.13
	Quercetin	10	0.11 ± 0.01
		20	1.00 ± 0.06
5% *w*/*w*	Extract	10	0.16 ± 0.01
		20	1.15 ± 0.07
	Quercetin	10	0.19 ± 0.01
		20	1.37 ± 0.08

**Table 4:** Parameters and correlation coefficients of each equation from the kinetic models of the zein microstructures loaded with quercetin or extract. Samples marked with * did not fit any model due to their fast core release.

	Korsmeyer–Peppas			Weibull		
Sample	*K_k_* (min^−1^)	*n*	R^2^	*Td* (min)	*β*	R^2^
10Z1Q	*					
10Z1EX	*					
20Z1Q	0.495	0.158	0.419	5.83	0.333	0.524
20Z1EX	0.309	0.275	0.446	15.57	0.408	0.352
10Z5Q	0.354	0.194	0.921	18.34	0.524	0.933
10Z5EX	*					
20Z5Q	0.151	0.354	0.985	63.32	0.486	0.963
20Z5EX	0.346	0.372	0.283	0.82	0.219	0.557

## Data Availability

The original contributions presented in the study are included in the article, further inquiries can be directed to the corresponding author.
